# Transportability Analyses in Comparative Effectiveness Research: A Conceptual Framework and Methodological Principles Endorsed by the International Society for Pharmacoepidemiology

**DOI:** 10.1002/pds.70396

**Published:** 2026-05-17

**Authors:** Blythe Adamson, Wei Liu, Stephen Duffield, Anouk Déruaz‐Luyet, Dimitri Bennett, Montse Soriano Gabarró, Mehmet Burcu, Daniela Claudia Moga, Sarah Welby, Tianze Jiao, Xuerong Wen, Grammati Sarri

**Affiliations:** ^1^ The Comparative Health Outcomes, Policy and Economics Institute School of Pharmacy, University of Washington Seattle Washington USA; ^2^ Infectious Economics, LLC New York New York USA; ^3^ Office of Surveillance and Epidemiology, Center for Drug Evaluation and Research, US Food and Drug Administration Silver Spring Maryland USA; ^4^ National Institute for Health and Care Excellence London UK; ^5^ Boehringer Ingelheim International GmbH Ingelheim am Rhein Germany; ^6^ Takeda Development Center Americas, Inc. Cambridge Massachusetts USA; ^7^ Independent Researcher Potsdam Germany; ^8^ Merck & Co., Inc. Rahway New Jersey USA; ^9^ University of Kentucky Lexington Kentucky USA; ^10^ GSK Wavre Belgium; ^11^ University of Florida Gainesville Florida USA; ^12^ University of Rhode Island Kingston Rhode Island USA; ^13^ Cytel, Inc London UK

**Keywords:** causal inference, comparative effectiveness research, decision‐making, external validity, lifecycle management, transportability

## Abstract

**The Problem:**

Transportability considerations are increasingly important to answer research questions in comparative effectiveness research (CER) to support the transfer of evidence on medicinal products across countries or settings. As drug development costs rise and healthcare systems and professionals face economic and resource pressures, leveraging existing data and evidence generated across borders or settings can improve efficiency and inform decisions in product development and decision‐making (regulatory, health technology assessment [HTA] and clinical care). Differences in population characteristics, healthcare systems, and data availability, including coding discrepancies, may present significant challenges to the transportability of CER evidence. Without rigorous methodological approaches, the utility of transportability analyses is limited.

**What we Did:**

This article provides a structured framework for considering transportability exercises in CER analyses. We outlined key methodological principles, when and why to use transportability exercises in CER, feasibility assessments including effect modifier identification and causal inference techniques such as weighting, outcome regression, and combined methods to guide transportability analytical approaches in CER. By synthesizing existing literature and expert insights, we identified opportunities and trade‐offs in applying transportability methods to support decisions across the product lifecycle.

**Strategies to Disseminate and Facilitate Use:**

To enhance the adoption of transportability analyses, we proposed best practices for researchers, regulators, HTA bodies, and industry stakeholders. These include early engagement with regulatory agencies and HTA bodies, transparent documentation of data assumptions, quality, fitness, and comparability assessments while ensuring robust analytical approaches. We also emphasized the need for standardized reporting guidelines and cross‐country collaborations to validate transportability methods in real‐world settings and communicate uncertainty in transported evidence.

**Conclusions:**

Transportability analyses offer a powerful tool for extending the applicability of CER findings across healthcare systems, improving evidence generation efficiency, and supporting global drug development and evaluation. By implementing best practices that promote a rigorous and transparent approach to the design and conduct of such analyses, stakeholders can maximize the value of transported treatment effects while ensuring scientific rigor and decision‐making relevance. Future research should focus on empirical testing and validation of transportability methods targeting different questions across the product lifecycle and the development of harmonized regulatory and HTA methodological standards. “This manuscript is endorsed by the International Society for Pharmacoepidemiology (ISPE).” Official Endorsement was received on 5/13/26.

## Key Points

- This manuscript establishes a clear, stepwise framework for transportability exercises, enabling reliable extension of study results across populations and settings.
- It promotes transparency, standardized reporting, and early engagement with decision‐makers to build confidence in transported evidence.
- The guidance supports better healthcare decisions globally, especially where local data are limited or before treatment uptake in routine care, by harmonizing methods and encouraging international collaboration.

## Plain Language Summary

This report explains how researchers can use data from one country or healthcare setting to help answer important questions about how well medicines work in other places where local data may be limited. The authors present a clear, step‐by‐step framework for “transportability analyses,” which are special methods that help extend research findings on medicines from one population to another (e.g., from patients in one country to similar patients in a different country). They highlight the need for careful planning, transparent reporting, and early discussions with decision‐makers like regulators and health technology assessors. It also describes best practices for choosing and analyzing data, checking assumptions, and communicating uncertainty. By following these best practices, researchers and policymakers can make better use of existing evidence to inform local decisions about medicines, especially in places where running new studies is difficult. This approach can help improve healthcare decisions worldwide by making sure that evidence is both scientifically rigorous and relevant to local needs.

## 1 Introduction

Across a medicinal product's lifecycle, the estimation of comparative effectiveness versus treatments used in clinical care remains fundamental to assess the product's value and inform policy decision‐making from clinical development through to post‐marketing authorization. Although randomized controlled trials (RCT) remain the gold standard of evidence to assess the efficacy of a medicinal product, their lack of broader, real‐world population representation limits the external validity of findings [1, 2, 3]. In an era of globally interconnected healthcare systems and amid mounting economic pressures across the healthcare landscape, there is growing interest in methods and analytical applications that can efficiently expand and transfer findings across study designs, populations, settings, and countries.

Comparative‐effectiveness research (CER) aims to inform decisions either early in product development or later in healthcare decision‐making by providing evidence on the relative benefits and risks of different medicinal products. Real‐world evidence (RWE) generated using pharmacoepidemiologic methods and analytical tools has gained momentum in terms of bridging the evidence gap between controlled trial environments (high internal validity) and varying populations in clinical practice (high external validity). Thus far, RWE guidance including the FDA's RWE framework and subsequent draft guidance on data standards and EHR/claims‐derived data [4, 5], the EMA's DARWIN EU framework [6, 7], the NICE RWE Framework [8], the CDA‐AMC reporting guidance [9], and the ENCePP methodological guide [10] has primarily focused on issues around data quality, fitness‐for‐purpose, transparency, and robust study designs leveraging real‐world data (RWD) [11, 12], with less emphasis on analytical opportunities to address external validity biases and transfer knowledge across settings. The true underlying causal effect typically varies with the definition of the selected target population, and applicability of findings from one country, region, or population to another remains a persistent challenge for stakeholders worldwide [13].

The challenge of extending insights and inferences beyond the original study samples under investigation has given rise to the topics of generalizability and transportability analyses as to enhance external validity in pharmacoepidemiology [14, 15]. Limitations in external validity have long been acknowledged as a concern in both randomized controlled trials and observational studies [9, 16]. We define *generalizability* as relating to whether inferences from a study can be extended to a target population from which the study participants were sampled; we define *transportability* as relating to whether inferences can be extended to a separate (external) population from which the study sample was not derived. In practice, source and target populations typically exhibit partial rather than complete overlap; transportability analyses apply whenever the source sample is not itself drawn from the target population and require positivity of selection over the covariate support of effect modifiers rather than strict disjointness of the two populations [17, 18]. A glossary of terms used in this paper is provided in Box 1.

BOX 1 **Glossary of Key Terms.**

**Average Treatment Effect (ATE):** A measure that represents the average effect of a treatment or intervention on a population. It contrasts outcomes between a group that received the treatment and a group that did not.

**Average Treatment Effect on the Treated (ATT):** A measure that represents the average impact of a treatment or intervention on those who actually received it. It focuses specifically on the subgroup of individuals who were exposed to the treatment, rather than the entire population.

**Conditional Exchangeability:** In relation to the transportability of relative effects, this is the assumption that all effect modifiers differing between populations are measured and adjusted for; also referred to as S‐admissibility.

**Consistency:** A foundational identifiability assumption stating that the observed outcome for an individual under the treatment received equals that individual's potential outcome under that treatment (i.e., there are no multiple versions of treatment) [19].

**Directed Acyclic Graphs (DAGs):** Visual representations of causal relationships and selection mechanisms between populations that help identify variables requiring adjustment in transportability analyses.

**Doubly‐Robust Methods:** Statistical approaches that integrate both weighting and outcome regression, offering protection against misspecification of either model, including targeted maximum likelihood estimation (TMLE) and augmented weighting estimators.

**Effect Modifiers:** Variables that influence the magnitude or direction of treatment effects across different populations or settings.

**External Validity:** The extent to which causal inferences from a study apply to populations, settings, or times beyond those represented in the study sample *(*“how well results generalize or transport to other contexts?”). External validity encompasses both generalizability (when the study sample is drawn from the target population) and transportability (when the study sample and target population are fully or partially distinct) [17, 20].

**Generalizability:** Extending causal inferences from a study sample to a broader target population of which the sample is a subset. In contrast to transportability, the study and target populations overlap by construction; positivity of selection need only be bounded away from zero (not from one) [17].

**Internal Validity:** The unbiased estimation of treatment effects within the study, focusing on the correctness of inferences in the original study context (“an effect estimate is unbiased for the causal treatment effect in the population from which the sample is a simple random sample”) [17].

**Matching‐Adjusted Indirect Comparison (MAIC):** An indirect‐treatment‐comparison method that applies method‐of‐moments calibration weighting to reweight the individual patient data of one study so that its covariate moments match reported summary statistics from a comparator study. In the transportability setting, the equivalent method‐of‐moments/entropy‐balancing estimator is more commonly applied to reweight source IPD to match target summary statistics [21, 22].

**Method‐of‐Moments / Entropy Balancing:** A family of calibration‐weighting estimators that derive weights so that reweighted covariate moments in the source sample exactly match specified target summary statistics. Particularly useful in transportability analyses when individual patient data are available in the source but only aggregate summaries are available in the target. Entropy balancing is mathematically equivalent to method‐of‐moments calibration under standard implementations [22].

**Outcome Regression Methods (in Transportability Analysis):** Statistical approaches that involve fitting models for the conditional outcome expectation using study data and then standardizing predictions to the target population's characteristics.

**Population Average Treatment Effect (PATE):** The causal estimand defined as the expected difference in counterfactual outcomes if every individual in the target population were to receive the intervention versus the comparator. PATE is a population‐level quantity and is not directly observable; transportability analyses aim to produce estimators of PATE in a target population using data from a source study [19].

**Positivity:** Assumption requiring non‐zero probability of study inclusion across all strata of effect modifiers, ensuring representation of all relevant subgroups.

**Real World Data (RWD):** Routinely collected data relating to patient health status and/or the delivery of health care routinely collected from a variety of sources, such as electronic health records (EHRs), claims and billing activities, product and disease registries, and data from other sources like mobile devices. Alternatively defined as data captured outside of the highly controlled trial environment (including other observational, even prospective, study designs conceptually similar to registries).

**Real‐World Evidence (RWE):** Information or evidence derived from the analysis of real‐world data (RWD), including treatment effectiveness in routine clinical practice to bridge the gap between controlled trial environments and diverse patient populations.

**Sample Average Treatment Effect (SATE):** The treatment effect in the finite source study sample. SATE is an estimator of the underlying population‐level estimand (typically PATE) under the assumptions outlined in this framework, not a distinct population‐level estimand [19].

Selection Diagrams: Extensions of directed acyclic graphs (DAGs) that incorporate selection nodes (S‐nodes) to represent variables whose distributions are expected to differ between the source study population and the target population. Selection diagrams provide a formal framework for identifying the set of variables requiring adjustment in transportability analyses.

**Target Population:** The group to whom researchers wish to apply study results; the population of interest for decision‐making.

**Transportability (property):** A property of a causal estimate or study, describing the extent to which an effect estimated in a source population can be extended, under stated assumptions, to a distinct target population that is external to the source. Transportability is typically operationalized through identification conditions on effect modifiers, selection mechanisms, and positivity [17], [23].

**Transportability Analyses (method):** The set of formal quantitative methods used to estimate causal effects in a target population using data from a source study, adjusting for differences in the distribution of effect modifiers between populations. Common approaches include inverse‐odds‐of‐sampling weighting, outcome regression with standardization, calibration weighting (method‐of‐moments / entropy balancing), and doubly‐robust estimators [20, 24].

**Transportability Feasibility Assessments:** Qualitative and quantitative evaluations, conducted prior to formal transportability estimation, of whether the identification conditions (S‐admissibility, positivity of selection, SUTVA for study selection) and data requirements (measured effect modifiers, covariate‐support overlap) are plausibly met for a given source‐target pair.

**Weighting Methods:** Statistical approaches that transport effect estimates by adjusting for differences in effect modifier distributions between populations, including inverse odds of sampling weights and calibration approaches.

The need for maximizing the value of the totality of evidence generated for a medicinal product, beyond evidence provided exclusively by clinical trials, has been increasingly recognized by regulatory agencies, health technology assessment (HTA) bodies, sponsors, and clinicians. When local data are sparse, unavailable, or lack properties to ensure trustworthy analyses, properly conducted transportability analyses can provide critical insights to inform causal effect estimates for relevant target populations [25]. This is particularly valuable for studies involving rare diseases, underserved populations, evidence re‐assessment, or restrictions due to strict local data privacy legislations. As access to clinical trials may be restricted in some countries due to capacity or infrastructure barriers [26], decision‐makers are increasingly uncertain about the ability to adjust for differences in key characteristics between the original data source and the target population of interest.

There is growing standardization of methodologies to adjust for differences in population demographic and clinical characteristics to enhance transportability [27]. Despite the potential benefits of transporting treatment effects across settings, regions, or countries, there remains uncertainty in unmeasured confounding factors related to healthcare system differences that needs to be further explored. Additionally, differences in treatment patterns and sequences between settings can introduce heterogeneity in findings and, in some cases, prevent the feasibility of transportability analyses [4, 28].

Previous publications have summarized the technical information and the uses of the transportability methods in a particular decision context (e.g., HTA) [29, 30, 31, 32, 33] and previous scoping reviews have presented studies applying transportability methods [34] but a conceptual framework that seeks to holistically address methodological considerations is lacking.

This paper aims to provide a conceptual, stepwise framework outlining data and methods considerations and best practices for researchers and decision‐makers for the applicability of transportability exercises of health technologies. Applying this framework can enhance the external validity of findings through the appropriate application of transportability analyses in CER, ultimately contributing to more efficient and effective healthcare decision‐making worldwide. “This manuscript is endorsed by the International Society for Pharmacoepidemiology (ISPE).” Official Endorsement was received on 5/13/26.

## 2 Methodological Approach

A broad, targeted literature review (TLR) on transportability methods, applications and case studies conducted in March 2025 (identified publications are presented in Table S1) and a snowballing approach to identify key publications were used to inform this guidance paper. The viewpoints of this paper were further shaped through discussions among ISPE CER Special Interest Group (SIG) working group members, the ISPE RWE Collaborative SIG, and external collaborators (research, industry, regulatory, HTA) and were endorsed via wider ISPE membership review.

The group critically assessed the methodological applications of transportability analyses in CER. Subsequently, the group developed a conceptual framework and a set of best practices to guide researchers undertaking transportability analyses in the context of pharmacoepidemiology and healthcare decision‐making, which was widely reviewed by ISPE members as part of the ISPE endorsement process. Previous discussions about transportability have mainly focused on transporting evidence between countries. We expand the scope beyond country differences to cover different healthcare practices (hospitals, community centers), regions within the same country, and decision‐making contexts (from regulatory to HTA).

An overview of the publications identified by the TLR is presented in Table S1. Most of these publications explored transportability and causal inference in CER (linked to the decision making at regulatory and HTA phases) relevant to the focus of this manuscript. Furthermore, in Figure S2 we describe potential applications of transportability analyses across the product lifecycle. We note that applications in exploratory, non‐registrational trials (e.g., Phase 1 MTD/OBD studies or single‐arm Phase 2 dose‐finding trials) are substantially constrained by the absence of randomization, limited internal validity of the source estimate, and small sample sizes, and remain largely theoretical. The framework is most actionable in post‐authorization, HTA, and cross‐country regulatory comparative‐effectiveness decision contexts.

The development of our methodological framework expands on prior systematic literature reviews by VanderWeele and Hernán [30], and Hernán and Robins [29].

### 2.1 Transportability Conceptual Framework and Methodological Principles

**FIGURE 1:**
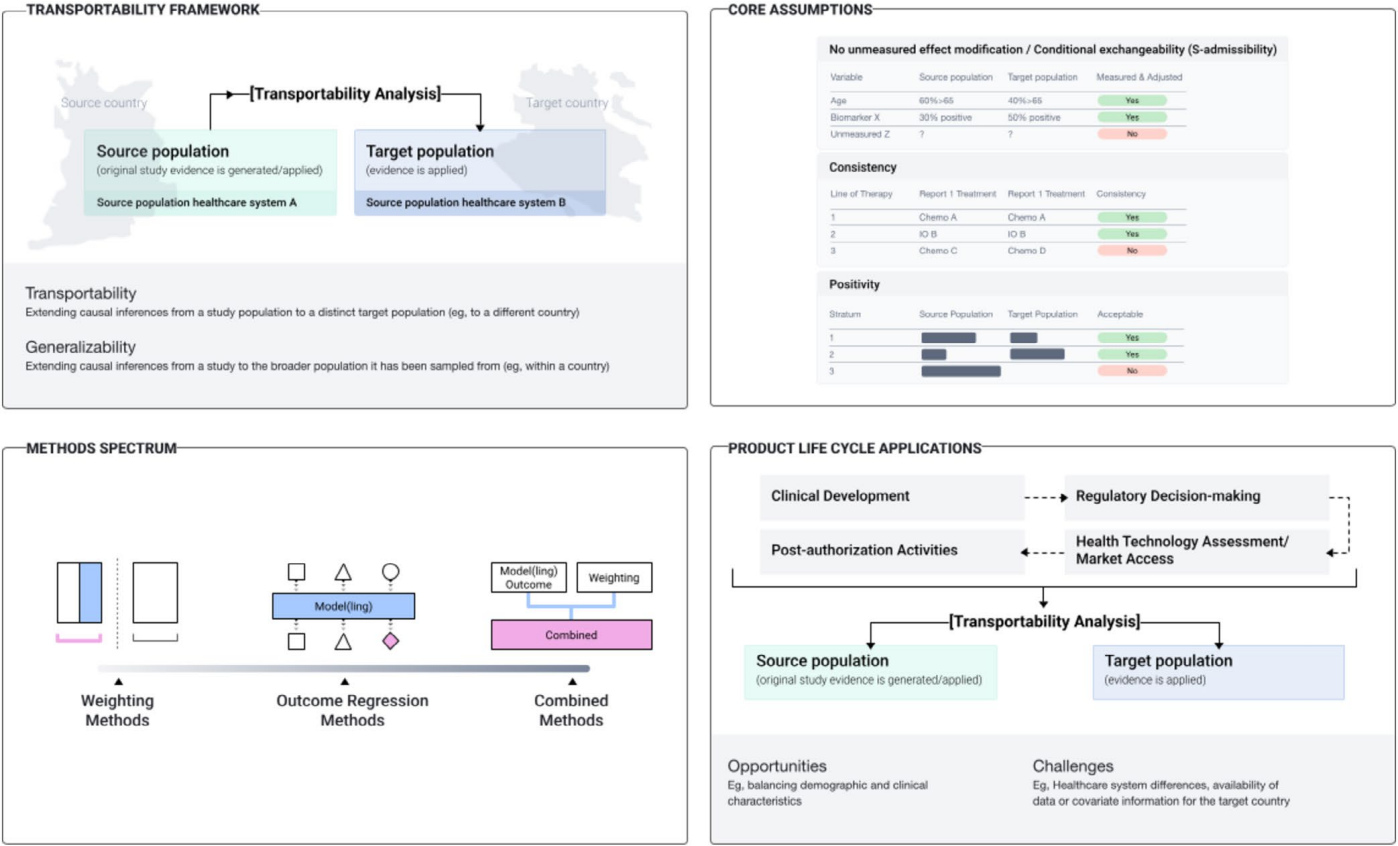
Conceptual diagram of transportability exercises.

A conceptual diagram of transportability topics is presented in Figure 1. A systematic, thorough, and transparent approach is required to apply transportability exercises including systematic evidence identification, transportability feasibility (qualitative) assessments and transportability analyses by applying formal quantitative methods for producing context‐specific (or local) estimates of comparative effectiveness and safety estimates of medicinal products by extending an effect estimate across populations after differences adjustment in effect modifiers. At the core of transportability analysis lies the causal inference methodology and its tools, which provides a formal theoretical framework for estimating treatment effects across different populations and settings. The simplest application of transportability analyses aims toward answering the following question of external validity: “If the same study was conducted in a different population (e.g., in a different country) with similar inclusion/exclusion criteria, what would the effectiveness and safety results for the treatment have been?*”*.

The question underpinning transportability exercises is intuitive; if there are certain characteristics (e.g., patient demographics, disease severity, healthcare systems) that modify the effectiveness and safety of an intervention and the distribution of these characteristics varies across settings (or countries), measuring and accounting for these characteristics would allow estimation of the effectiveness of an intervention in an external setting (or another country) without the presence of context‐specific outcome data.

Transportability analyses specifically address the challenge of extending causal inferences from a source study to a distinct target population (external population) and strive for both internal and external validity. While generalization and transportability involve distinct conceptual considerations regarding differences between study and target populations, the analytical methods used to address each are closely related. Our framework adopts this applied interpretation: while Bareinboim and Pearl's formalization of transportability vs. generalizability through strict set‐theoretic relations is pedagogically useful, in practice these concepts operate on a continuum, with the required condition being covariate‐support overlap in the distribution of key effect modifiers. Throughout the manuscript, when needed, we note these differences in the assumptions and methodological considerations required for transportability and generalizability.

### 2.2 Transportability Framework

**FIGURE 2:**
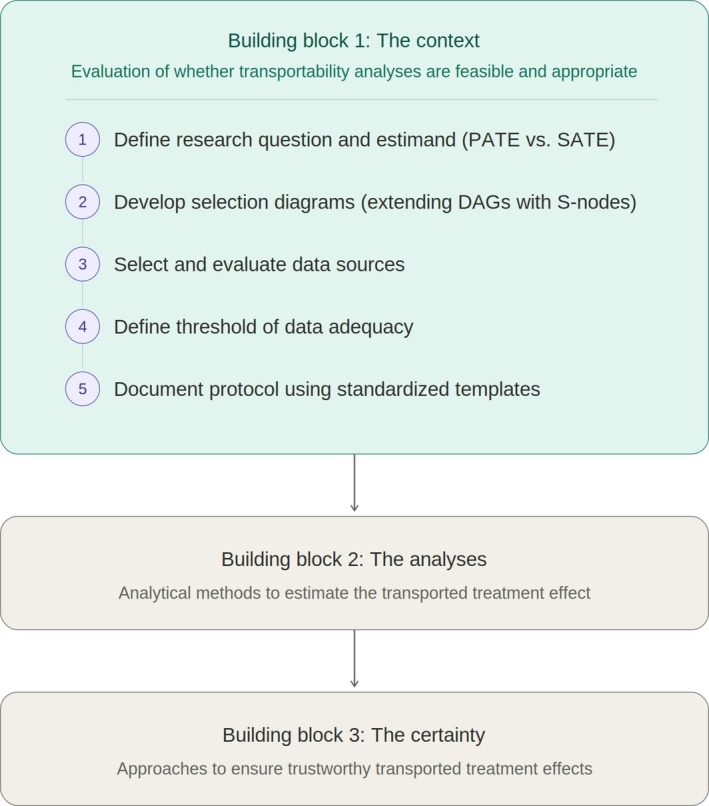
The building blocks of transportability exercises in comparative‐effectiveness research.

Our proposed transportability framework consists of three main building blocks as depicted in Figure 2 with detailed sequential steps captured in each of these blocks. Following the description of the three building blocks, we outline potential applications of transportability analyses to different phases of the medicinal product lifecycle.

#### 2.2.1 Building Block 1: Evaluation Processes of Whether Transportability Exercises Are Feasible and Appropriate or Not (“The Context”)

As a first step, a research question needs to clearly define the purpose of the transportability exercises and the characteristics of each of the following critical concepts: the source sample (the analysis set for the original study), the source population (the population targeted by the original study), and the target population (the population to whom we wish to apply the results).

We distinguish throughout between the causal estimand, the target quantity defined at the population level (most commonly the population average treatment effect, PATE), and estimators of that estimand computed from finite samples. The sample‐average treatment effect (SATE) from the source sample is one such estimator; transportability analyses aim to construct estimators of the PATE for the target population under the assumptions described below. The choice of estimand is fundamental to transportability exercises by defining the type of transported average treatment effect (tATE). ICH E9(R1) outlines statistical principles for defining estimands in clinical trials, focusing on five attributes: population, treatment, endpoints, handling of intercurrent events, and the population‐level summary measure [35]. This framework was developed primarily for randomized trials conducted in controlled settings. Marginal summary measures (e.g., risk differences, marginal odds ratios) and conditional summary measures (conditional on covariates) can yield divergent transported estimates even under the same effect‐modifier adjustment set; Remiro‐Azócar et al. (2025) [36] provide a formal treatment of when these measures are and are not compatible across studies. Valid transportability therefore requires alignment between the estimand (marginal versus conditional) and the analytical approach used to estimate and transport it. Beyond distributional differences in effect modifiers, cross‐setting transportability can be complicated by healthcare‐system‐specific handling of intercurrent events—for example, the availability or sequencing of subsequent therapies may differ between the source and target settings. Treatment‐policy estimands implicitly average treatment effects over care pathways that are specific to the source setting; when these pathways differ across settings, transported estimates may not correspond to an estimand of decision relevance in the target setting, and the transportation of model‐based hypothetical estimands (e.g., envisioning a target setting in which a subsequent therapy is unavailable) remains an open methodological question [37].

In contrast, specifying estimands for studies using real‐world data (RWD), including real‐world evidence studies, presents additional challenges that are closely related to issues of transportability [19, 38].

RWD studies typically involve heterogeneous populations that may differ systematically from randomized trial populations with respect to demographic characteristics, disease severity, treatment access, and care delivery. Treatment strategies in RWD are often dynamic and incompletely defined, involving treatment switching, discontinuation, or variable adherence, which further complicates specification of the treatment component of the estimand [37]. Intercurrent events such as competing events or changes in standard of care may also occur more frequently and under different mechanisms than in the controlled environment of clinical trials, affecting the assumptions required for transportability exercises.

Explicit and transparent estimand specification in RWD studies is therefore essential for clarifying target causal effects and assessing the plausibility of transporting effect estimates to external populations.

For Step 2, the assumptions (conditions) for transportability exercises have important implications for variable selection. The causal relationships between patient and intervention characteristics, as well as contextual factors (e.g., type of healthcare systems, clinical practice and access patterns including impact of socioeconomic factors) are critically relevant [27]. A commonly used strategy is to identify a sufficient adjustment set by blocking all paths from selection (S) nodes to the outcome in a selection diagram, an extension of directed acyclic graphs (DAGs) developed for transportability exercises. Of note, probability distributions are not applied to selection nodes, meaning that they cannot be influenced by other variables in the graph. Although this approach ensures sufficiency, the resulting adjustment set is not necessarily minimally sufficient. In particular, it may include variables that do not threaten transportability—such as those that do not modify the effect on the estimand of interest—thereby reducing precision by inflating the variance of effect estimates. Moreover, blocking all such paths may necessitate adjustment for mediators, which complicates interpretation and introduces additional assumptions concerning the transportability of mediated effects.

Specification of the effect measure scale (e.g., additive) can yield smaller minimally sufficient adjustment sets with improved precision compared with approaches based solely on graphical criteria. For example, when transporting the risk difference, adjustment is required only for variables that both modify the risk difference and differ in the distribution between the source and target populations [39, 40]. However, variable selection based on effect measure modification cannot be determined from DAGs or selection diagrams alone, as these tools encode causal structure but not assumptions about effect scale (marginal or conditional effects) or collapsibility [41], information that may not be available. Additionally, variables that are not strict effect modifiers may still be required to transport non‐collapsible measures, such as odds ratios or hazard ratios. In these transportability exercises, variable selection is often influenced by pragmatic considerations, including data availability, recency, and sample size constraints [39, 42]. We recommend a comprehensive background literature review using reproducible criteria to inform the development of selection diagrams.

All transportability exercises rely on four key assumptions (Figure 1): (1) internal validity of the original study, e.g., no unmeasured confounding, positivity of treatment assignment and the stable unit treatment value assumptions [SUTVA] (these assumptions are met by design for randomized studies but not for non‐randomized studies), (2) conditional exchangeability over selection (S‐admissibility), requiring that all effect modifiers differing between the study and the target population are measured and accounted for, (3) positivity of selection, requiring overlap in the distribution of effect modifiers in the study and target population (meaning that all effect modifiers in the target population are observed in the study), and (4) SUTVA for study selection, requiring no interference between subjects in the study and the target population and no differences in how outcomes are measured between settings [31, 32]. Researchers should refrain from conducting transportability analyses when key assumptions, such as conditional exchangeability, positivity, or the availability of essential effect modifiers, cannot be reasonably met due to data limitations or substantial differences in healthcare systems, patient populations, or treatment pathways between the source and target settings. Here we make recommendations to reflect the key assumptions described above. Of note, there are some distinct considerations in these assumptions that differentiate transportability from generalizability analyses; firstly, when testing for conditional exchangeability over selection in transportability analyses, the set of variables should not include those that distinguish the study from the target population (e.g., country), a requirement which is not applicable in generalizability analysis. Secondly, for the positivity of selection assumption to hold in transportability analyses, the estimated propensity to be in the study population need to be bounded away from the values of 0 and 1 ensuring overlap without exclusivity, whereas in generalizability analyses the propensity only needs to be bounded away from 0 since the study is a subset of the target population. For further technical details, we recommend the papers by Dahabreh et al. [14, 24, 43], Degtiar and Rose (2023) [17] and Elliott et al. [44].

In Step 3, the selection and evaluation of appropriate data sources (e.g., clinical trial data, electronic health records, claims data, and patient registries) are critical components of transportability exercises. Each data type presents unique challenges for cross‐settings or cross‐country comparisons. For successful transportability analyses, minimum data requirements include comprehensive documentation of essential effect modifiers (e.g., age, biomarker status, disease severity) and prognostic factors that may influence treatment outcomes. Contextual variables describing healthcare settings and bridging variables that facilitate linkages between populations are equally critical. As previously highlighted, researchers should specifically document and assess treatment pathways, the nature of the healthcare system, and the incidence and prevalence of the indication across jurisdictions when evaluating cross‐border data fitness for transportability analyses [28]. In the most common transportability setups, outcome data are required only in the source study, while the target population contributes distributional information on effect modifiers and other covariates in the adjustment set. Harmonization of variable definitions and coding systems for these covariates is therefore the principal data‐quality concern in the target. Standardization of outcome ascertainment, time frames, and follow‐up periods between source and target is relevant when target‐population outcome data are available (for benchmarking, validation, or joint analyses) [33] but is not a prerequisite for standard transportability estimation.

Data availability and governance introduce additional practical constraints. Even when individual patient‐level data (IPD) in principle exist in both source and target populations, privacy or data‐sharing restrictions frequently prevent cross‐border data movement and therefore preclude dataset merging on which conventional propensity‐score weighting depends. A common operational configuration is IPD in the source together with aggregate‐level summaries (e.g., covariate means, proportions, standard deviations) in the target. In such settings, calibration‐weighting estimators including method‐of‐moments estimators (used in matching‐adjusted indirect comparison, MAIC) and, more directly applicable to transportability, entropy balancing can be applied using source IPD and target summary statistics [27]. Methodological development for this IPD‐to‐aggregate scenario remains comparatively limited, and we flag it as a priority for future work.

Step 4 of 5 involves defining the threshold of data adequacy (“what is considered good enough?”). Given that, to an extent, possible differences in populations and settings are expected and that data availability is restricted by local protocols and network infrastructures, defining the threshold of data adequacy can be challenging for some analyses and dependent on the purpose of transportability exercises (what evidence gaps aim to cover). Setting up the “core” set of primary variables in selection diagrams and maximizing the knowledge from additional variables collected pre‐ and post‐intervention can facilitate decision‐making about the feasibility of transportability analyses, based on the hypothesized data‐generating process.

We recommend following steps 1–4, consistent with a previously developed ISPE‐endorsed framework, for an unbiased identification, selection and critical assessment of the evidence sources to best reflect the targeted population and its characteristics [45]. The availability of IPD from representative sources such as electronic health records will allow more trustworthy transportability analyses and facilitate the use of advanced methodologies to account for different types of biases, if these exist (selection, confounding, measurement). Therefore, it is highly recommended for transporting treatment effects. Attention is required when using electronic health records (EHRs) for transportability analyses given differences in structure and completeness across healthcare systems and these challenges may be disease specific [46]. Detailed guidance including previously published RWE frameworks on study design, planning and fit‐for purpose assessment for non‐randomized evidence including routinely collected patient data has been presented by different decision‐makers (FDA [4, 5], EMA [6, 7], NICE [8], CDA‐AMC [9]) and international organizations (ISPE [12], ENCePP [10], ISPOR [12]) and we encourage researchers undertaking transportability analyses to use standardized templates (e.g., RECORD [47], RECORD‐PE [47], DataSAT [8]) and guidance to inform and record these decisions.

Finally, for Step 5, we advise researchers to follow internationally recommended guidance and templates (EMA [6, 7], NICE [8], CDA‐AMC [9]) (such as the use of The HARmonized Protocol Template to Enhance Reproducibility (HARPER) [12], an output of a joint ISPE/ISPOR task force effort) to record details of the transportability study protocol and consider its publication in international platforms (European Network of Centres for Pharmacoepidemiology and Pharmacovigilance, ClinicalTrials.gov, RWE Transparency Initiative) [48].

The proposed conceptual process is also important to recognize situations where a formal transportability analysis should not be attempted. In such cases, attempting such analyses may yield misleading or biased results and undermine decision‐making validity. In lower‐stakes situations, or when the primary goal is hypothesis generation rather than regulatory or reimbursement decision‐making, it may suffice to transparently present descriptive differences between populations or settings. Expert input can be sought when needed to qualitatively assess the importance of observed differences, providing context for stakeholders. Additionally, researchers can leverage other external information such as published literature, disease registries, or benchmarking studies to compare outcomes across settings and triangulate evidence, even if formal adjustment is not feasible. In some cases, researchers may also consider data transferability as the direct use of real‐world data generated in another jurisdiction to address local evidence gaps as a complementary or alternative strategy when formal quantitative transportability of treatment effects is not feasible [28]. In some scenarios, particularly when critical data elements are missing or when there is substantial heterogeneity that cannot be addressed analytically, primary data collection in the target population may be necessary. This approach ensures that local context, treatment patterns, and patient characteristics are adequately captured to inform robust comparative effectiveness estimates. Ultimately, the decision to proceed with transportability analyses, or to pursue alternative methodological strategies, should be guided by a careful assessment of data quality, methodological feasibility, and the intended use of the evidence.

#### 2.2.2 Building Block 2: Analytical Methods to Estimate the Transported Treatment Effects

Statistical methods for transportability analyses are related to common confounding methods and fall into three categories: weighting methods, outcome regression methods, and combined approaches such as doubly robust approaches. Weighting methods can transport treatment effect estimates by conceptualizing a model for the probability of study membership (the inverse probability of target population membership); where individuals within a population receive weights creating a “pseudo‐population” with the same covariate distribution as a target population [20, 24]. Weighting approaches include modelling and calibration approaches. In the transportability context, inverse‐odds weighting (otherwise called “inverse odds of participation/sampling weights”) is a variation of weighting methods when the populations in the study and the target groups are completely separate; a weight equal to their covariate‐conditional inverse odds of membership in the study population is assigned to each individual so the weighted population has the same distribution of covariates in the adjustment set as the target population [31]. Previous publications have pointed out that attention is needed to ensure increase in covariate imbalance, after weighting, does not further worsen confounding. Outcome regression methods involve fitting models for the conditional outcome expectation to the study, then standardizing predictions to the target population's characteristics [27, 49]. This approach was employed in one transportability case study where pooled logistic regression models for overall survival were standardized to Canadian population covariate distributions to obtain transported survival probabilities from US data [50]. More recently, doubly‐robust methods were developed, integrating both weighting and outcome regression approaches, offering protection against model misspecification by generating consistent estimates provided that either the weighting or the outcome model is correctly employed, but not necessarily both [36]. Doubly robust methods include targeted maximum likelihood estimation (TMLE) and augmented inverse probability weighted estimators.

We identified several reviews that may aid researchers in specific analytical scenarios. The reviews by Cook et al. (2024) [51] and Webster‐Clark et al. (2025) [27] provide a comprehensive presentation of statistical and scientific topics concerning the interpretation and replicability of transportability analyses. The review by Dahabreh et al. (2022) [43] highlights transportability methods in specific circumstances where there is an absence of unmeasured confounding or when effect measures are modified by a relatively small number of measured baseline factors; when the control treatment in the trial (sample population) is the only treatment in use in the target population or there exists a variation in treatments available but a partial exchangeability assumption may hold. The review by Degtiar and Rose (2022) [17] is inclusive of real‐world evidence. A review of more complex methods for transporting causal effect estimates from trials to populations included Bayesian additive regression trees for both inverse probability of treatment weighting and prediction estimation that do not require specification of functional form or interaction [44].

In the context of HTA, an overview of transportability methods and their applications including case studies can be found in several publications [29, 30, 31, 32].

#### 2.2.3 Building Block 3: Approaches to Ensure Trustworthy Transported Treatment Effects (“The Certainty”)

As for every analytical approach, identifying and assessing the size and direction of bias(es), including a quantification of its impact on the validity of results is a key step in CER. Sensitivity analyses for effect modifiers not observed in the target population (relaxing inclusion criteria) investigating alternative assumptions in the analytical modelling and the selection of RWD sources are essential in transportability analyses. HTA guidance highlights the importance of estimating the direction, magnitude, and uncertainty of biases associated with measures of effect using different methods such as E‐value calculations, bias functions, and tipping point (bias quantification) analyses (please see example of bias analysis in the NICE RWE Framework) [8]. Recent EU HTA guidance has also proposed the use of shifted null hypotheses to test uncertainty concerns in comparative effectiveness although concerns are raised regarding its applicability in the HTA context [27]. Post hoc analyses in transportability analyses exploring the role of time‐period effects given potential clinical practice or access differences between the settings (or countries), differences in censoring patterns are necessary to assess the impact on data comparability. Missing data presents additional challenges in transportability analyses. Multiple imputation by chained equations has been employed in transportability studies to address potential bias from missing data [please see case studies included in the review by Levy et al. (2024) [29]]. Measurement error can be assessed through quantitative bias analyses that impute values for mismeasured variables. It is also possible to consider how the assumption of ignorability may be assessed from observed data and propose a sensitivity analysis under the failure of this assumption [25].

Collectively, the approaches described in this building block serve a unified purpose: establishing a layered evidence base that increases confidence in the credibility and robustness of transported treatment effects. No single analytical check can definitively establish the validity of a transportability analysis; rather, confidence is built incrementally through triangulation across complementary methods. Sensitivity analyses assess the potential impact of unmeasured effect modifiers, bias quantification methods (e.g., *e*‐values, bias functions, tipping‐point analyses) characterize the magnitude of bias needed to alter conclusions, and post hoc diagnostics evaluate the influence of time‐period effects, censoring patterns, and missing data on comparability. When the findings of a transportability analysis are consistent across these different angles of scrutiny, each probing a different threat to validity, the overall body of evidence is substantially strengthened. Conversely, when results are sensitive to assumptions or data limitations, this process transparently identifies where further investigation or caution in interpretation is warranted.

To maximize the impact of this multi‐pronged validation strategy, early and ongoing engagement with regulatory agencies and HTA bodies is essential. Decision‐makers can provide critical guidance on which sensitivity analyses and bias quantification approaches are most relevant to their evidentiary standards, what thresholds of robustness are expected, and how uncertainty in transported estimates should be reported and communicated. Initiating this dialogue at the study design stage, rather than after analyses are complete, helps ensure that the analytical plan is aligned with decision‐making requirements from the outset and reduces the risk that methodological choices will be deemed insufficient at the point of evidence appraisal. This recommendation is consistent with established RWE frameworks from NICE [8], EMA [6, 7], and the FDA [4, 5], which emphasize scientific advice and protocol review as mechanisms for aligning study design with regulatory expectations. NICE's RWE Framework is the only one to date that has a specific section on transportability. We consider early stakeholder engagement to be a foundational element of Building Block 3, as even the most rigorous validation analyses will have limited impact if they do not address the specific concerns and standards of the intended decision‐making audience.

## 3 Summary and Conclusions

This article presents a conceptual framework for conducting transportability exercises for CER, informed by both a targeted review of the methodological literature and the collective experience of a multistakeholder group of epidemiologists, data scientists, and decision makers. This framework is intended to bridge the gap between transportability theory and its practical application in CER by reflecting real‐world constraints in the relevance of such methodologies.

**FIGURE 3:**
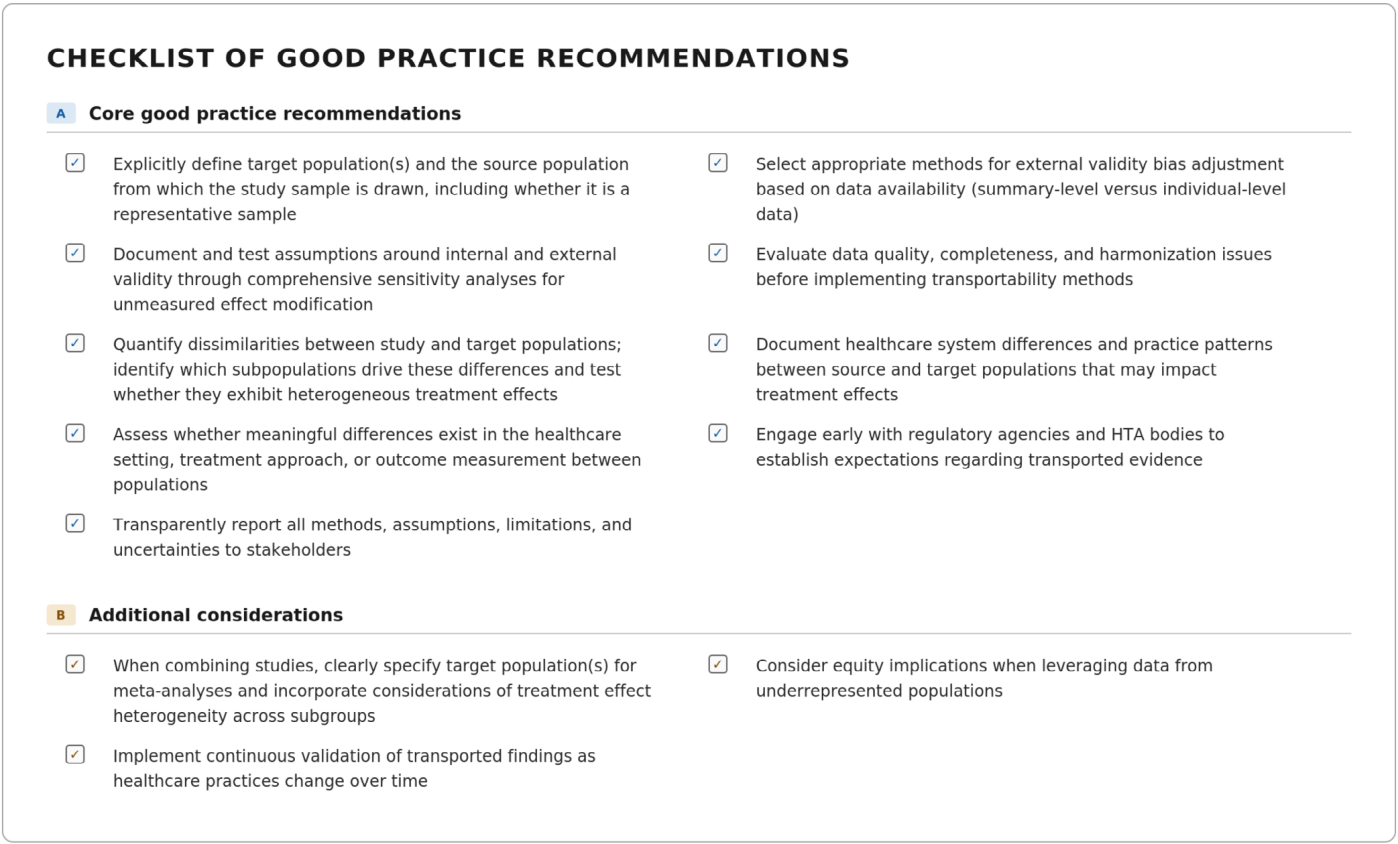
Checklist of Good Practice Recommendations for transportability exercises.

A checklist of good practices is provided in Figure 3. Transportability analyses, which extend causal inferences from a study to a distinct target population, offer significant value when local real patient data are unavailable before marketing authorization but there is insufficient or prohibitively expensive to generate or supplement RCT information to reflect varying real‐world populations. Regulatory acceptance of such evidence is inherently conditional and often depends on contextual factors such as unmet medical need, degree of innovation, endpoint relevance, and alignment with target‐setting standards of care. We have delineated key methodological principles, including the importance of identifying effect modifiers, satisfying conditional exchangeability assumptions, and employing appropriate statistical approaches such as weighting methods, outcome regression techniques, and doubly‐robust estimators. Our review identified phase‐specific opportunities and challenges, from enhancing trial design in pre‐approval phases to supporting post‐authorization effectiveness evaluations across diverse healthcare systems. Throughout, we emphasized the critical importance of transparent documentation of assumptions, comprehensive assessment of population and contextual factors differences, and rigorous validation analyses to address unmeasured effect modification and other sources of data uncertainty. Following well established RWE methodological standards and reporting requirements (including use of templates) is an integral part of these analyses. Prospectively assessing the need for transportability analyses (early in clinical development) and recognizing its potential to enhance evidence generation while acknowledging inherent limitations in the interpretation of findings is advisable. Early engagement with local regulatory and HTA bodies to assess the need and evidentiary requirements of transportability analyses is needed. Beyond the core framework, we identify additional considerations for researchers undertaking transportability analyses, including the specification of target populations when transported estimates are used in meta‐analyses, continuous validation of transported findings as clinical practice evolves, and the evaluation of equity implications when analyses leverage data from underrepresented populations (Figure 3, Section B). Researchers should prioritize the use of external evidence for transportability analyses from settings reflecting similar health care systems, with comparable patient epidemiological profiles and reflecting similar type HTA‐archetypes.

Future research should focus on extending and developing standardized approaches for quantifying dissimilarity between populations and validating transportability methods against prospectively collected data. Existing indices of population dissimilarity, such as the generalizability index proposed by Tipton (2014) [52], offer promising tools for quantifying the degree of covariate overlap between study and target populations, though their adoption in the real‐world data setting remains limited. We strongly advocate for systematic benchmarking studies comparing transported estimates with locally generated evidence across different disease areas, settings and healthcare systems. Such empirical validation is essential for establishing when transportability analyses can reliably substitute for local studies and for identifying contexts where significant effect heterogeneity limits transportability estimates. Finally, future research should more directly address data availability and access constraints as a central limiting factor in transportability analyses. Many proposed transportability methods rely on rich covariate information being jointly available—or at least conceptually in alignment—across source and target populations. Addressing these issues is critical for translating the theoretical appeal of transportability into routine applied use in healthcare decision‐making. Two additional open methodological questions warrant future work: (i) the transportation of model‐based hypothetical estimands across settings with differing intercurrent‐event pathways (e.g., differing subsequent‐therapy availability), which current treatment‐policy framings do not accommodate; and (ii) methodological development for the IPD‐to‐aggregate scenario, in which source IPD must be combined with target‐population summary statistics under data‐governance constraints that preclude dataset merging.

As globalization and digitalization of healthcare are evolving, advancing the science of transportability requires multi‐stakeholder collaboration between industry, regulatory agencies, HTA bodies, and academic researchers to harmonize methodological standards and identify opportunities for data partnerships and infrastructures that allow common data models application and standardized analyses. The recent EU regulation developments, such as the EU HTA Regulation and the EU Healthcare Data Space, may provide the ideal setting for safely testing the applications of transportability analyses across geographical settings within and beyond Europe.

## Funding

The authors have nothing to report.

## Disclosure

The contents are those of the authors and do not necessarily represent the official views of, nor an endorsement by NICE, the FDA/HHS, or the U.S. Government.

## Conflicts of Interest

D.B. is an employee of Takeda; A.D.‐L. is an employee of Boehringer Ingelheim International GmbH; M.S.G. holds shares of GSK; M.B. is an employee of Merck Sharp & Dohme LLC, a subsidiary of Merck & Co. Inc., Rahway, NJ, USA, and owns stock in Merck & Co. Inc., Rahway, NJ, USA; S.W. is an employee and holds shares of GSK; G.S. is an employee and holds shares of Cytel Inc. W.L. conducted the work in his personal capacity. The other authors declare no conflicts of interest.

## Supporting information


**Table S1:** Studies identified in literature review.
**Figure S2:** Potential product life‐cycle applications for transportability exercises in comparative‐effectiveness research.

## Data Availability

The data that supports the findings of this study are available in the Supporting Information of this article.

## References

[1] M. L. Berger , H. Sox , R. J. Willke , et al., “Good Practices for Real‐World Data Studies of Treatment and/or Comparative Effectiveness: Recommendations From the Joint ISPOR‐ISPE Special Task Force on Real‐World Evidence in Health Care Decision Making,” Pharmacoepidemiology and Drug Safety 26, no. 9 (2017): 1033–1039. 28913966 10.1002/pds.4297 PMC5639372

[2] J. M. Franklin , K. L. Liaw , S. Iyasu , C. W. Critchlow , and N. A. Dreyer , “Real‐World Evidence to Support Regulatory Decision Making: New or Expanded Medical Product Indications,” Pharmacoepidemiology and Drug Safety 30, no. 6 (2021): 685–693. 33675248 10.1002/pds.5222

[3] H. Yuan , M. S. Ali , E. S. Brouwer , et al., “Real‐World Evidence: What It Is and What It Can Tell us According to the International Society for Pharmacoepidemiology (ISPE) Comparative Effectiveness Research (CER) Special Interest Group (SIG),” Clinical Pharmacology and Therapeutics 104, no. 2 (2018): 239–241. 29733448 10.1002/cpt.1086

[4] S. V. Wang , S. Pinheiro , W. Hua , et al., “STaRT‐RWE: Structured Template for Planning and Reporting on the Implementation of Real World Evidence Studies,” BMJ 372 (2021): m4856. 33436424 10.1136/bmj.m4856 PMC8489282

[5] S. V. Wang , A. Pottegård , W. Crown , et al., “HARmonized Protocol Template to Enhance Reproducibility of Hypothesis Evaluating Real‐World Evidence Studies on Treatment Effects: A Good Practices Report of a Joint ISPE/ISPOR Task Force,” Pharmacoepidemiology and Drug Safety 32, no. 1 (2023): 44–55. 36215113 10.1002/pds.5507 PMC9771861

[6] M. A. Hernán and T. J. VanderWeele , “Compound Treatments and Transportability of Causal Inference,” Epidemiology 22, no. 3 (2011): 368–377. 21399502 10.1097/EDE.0b013e3182109296 PMC3805254

[7] I. J. Dahabreh , S. E. Robertson , J. A. Steingrimsson , E. A. Stuart , and M. A. Hernán , “Extending Inferences From a Randomized Trial to a New Target Population,” Statistics in Medicine 39, no. 14 (2020): 1999–2014. 32253789 10.1002/sim.8426

[8] J. Pearl and E. Bareinboim , “External Validity: From Do‐Calculus to Transportability Across Populations,” Statistical Science 29, no. 4 (2014): 579–595.

[9] A. Steckler and K. R. McLeroy , “The Importance of External Validity,” American Journal of Public Health 98, no. 1 (2008): 9–10. 18048772 10.2105/AJPH.2007.126847 PMC2156062

[10] Policy DMIfH , “International Harmonization of Real World Evidence Standards Dashboard,” 2025, accessed 16 April 16 2025, https://healthpolicy.duke.edu/projects/international‐harmonization‐real‐world‐evidence‐standards‐dashboard.

[11] EFPIA , “60,000 Fewer Clinical Trial Places for Europeans, Despite Global Surge in Research Projects,” European Federation of Pharmaceutical Industries and Associations, January 1, 2025.

[12] M. Webster‐Clark , A. Breskin , E. D. Duchesneau , and K. E. Rudolph , “Transporting: What Is It and How Do You Do It?,” Current Epidemiology Reports 12 (2025): 12. 10.1007/s40471-025-00374-6 PMC13038309 41923783

[13] FDA , “Considerations for Generating Clinical Evidence From Oncology Multiregional Clinical Development Programs; Draft Guidance for Industry,” Food and Drug Administration, September 17, 2025.

[14] A. Jaksa , P. J. Arena , K. K. Chan , R. H. Ben‐Joseph , P. Jónsson , and U. B. Campbell , “Transferability of Real‐World Data Across Borders for Regulatory and Health Technology Assessment Decision‐Making,” Frontiers in Medicine 16, no. 9 (2022): 1073678. 10.3389/fmed.2022.1073678 PMC9709526 36465931

[15] N. S. Levy , P. J. Arena , T. Jemielita , et al., “Use of Transportability Methods for Real‐World Evidence Generation: A Review of Current Applications,” Journal of Comparative Effectiveness 13, no. 11 (2024): e240064. 10.57264/cer-2024-0064 PMC11542082 39364567

[16] Q. Vuong , R. K. Metcalfe , A. Ling , B. Ackerman , K. Inoue , and J. J. Park , “Systematic Review of Applied Transportability and Generalizability Analyses: A Landscape Analysis,” Annals of Epidemiology 104 (2025): 61–70. 40064249 10.1016/j.annepidem.2025.03.001

[17] A. J. Turner , C. Sammon , N. Latimer , et al., “Transporting Comparative Effectiveness Evidence Between Countries: Considerations for Health Technology Assessments,” PharmacoEconomics 42, no. 2 (2024): 165–176. 37891433 10.1007/s40273-023-01323-1 PMC10811184

[18] G. Scelo , D. Zugna , M. Popovic , K. Strandberg‐Larsen , and L. Richiardi , “Transporting Results in an Observational Epidemiology Setting: Purposes, Methods, and Applied Example,” Frontiers in Epidemiology 4 (2024): 1335241. 38456074 10.3389/fepid.2024.1335241 PMC10910888

[19] FDA , “Guidance Document. Use of Real‐World Evidence to Support Regulatory Decision‐Making for Medical Devices. *Guidance for Industry and Food and Drug Administration Staff*,” Food & Drug Administration, 2017.

[20] I. Degtiar and S. Rose , “A Review of Generalizability and Transportability,” Annual Review of Statistics and Its Application 10, no. 1 (2022): 501–524.

[21] J. E. Signorovitch , E. Q. Wu , A. P. Yu , et al., “Comparative Effectiveness Without Head‐to‐Head Trials: A Method for Matching‐Adjusted Indirect Comparisons Applied to Psoriasis Treatment With Adalimumab or Etanercept,” Pharmacoeconomics 28, no. 10 (2010): 935–945. 20831302 10.2165/11538370-000000000-00000

[22] K. P. Josey , S. A. Berkowitz , D. Ghosh , and S. Raghavan , “Transporting Experimental Results With Entropy Balancing,” Statistics in Medicine 40, no. 19 (2021): 4310–4326. 34018204 10.1002/sim.9031 PMC8487904

[23] E. Bareinboim and J. Pearl “A General Algorithm for Deciding Transportability of Experimental Results,” Journal of Causal Inference 1, no. 1 (2013): 107–134.

[24] RECORD , “What is RECORD?,” 2019, https://www.record‐statement.org/.

[25] S. Kent , P. Mpofu , S. Duffield , et al., “Evaluating Transportability of Overall Survival Estimates From US to UK Populations Receiving First‐Line Treatment for Advanced Non‐Small Cell Lung Cancer: A Retrospective Cohort Study,” BMJ Open 14, no. 12 (2024): e085722. 10.1136/bmjopen-2024-085722 PMC11628978 39645255

[26] F. Manke‐Reimers , V. Brugger , T. Bärnighausen , and S. Kohler , “When, Why and How Are Estimated Effects Transported Between Populations? A Scoping Review of Studies Applying Transportability Methods,” European Journal of Epidemiology 40, no. 3 (2025): 255–273. 40249515 10.1007/s10654-025-01217-w PMC12137380

[27] ICH , “ICH Harmonised Guideline. Addendum on Estimands and Sensitivity Analysis in Clinical Trials to the Guideline on Statistical Principles for Clinical Trials ICH E9(R1) 2019”.

[28] A. Remiro‐Azócar , “Transportability of Model‐Based Estimands in Evidence Synthesis,” Statistics in Medicine 43, no. 22 (2024): 4217–4249. 39550630 10.1002/sim.10111

[29] M. A. Hernán and J. M. Robins , Causal Inference: What If (Chapman & Hall/CRC, 2020).

[30] T. J. VanderWeele and M. A. Hernán , “Causal Inference Under Multiple Versions of Treatment,” Journal of Causal Inference 1, no. 1 (2013): 1–20. 25379365 10.1515/jci-2012-0002 PMC4219328

[31] J. Chen , D. Scharfstein , H. Wang , et al., “Estimands in Real‐World Evidence Studies,” Statistical Biopharmaceutical Research 16 (2023): 1–25.

[32] K. E. Rudolph , N. T. Williams , E. A. Stuart , and I. Díaz , “Improving Efficiency in Transporting Average Treatment Effects,” Biometrika 112, no. 3 (2025): asaf027. 40800216 10.1093/biomet/asaf027 PMC12338304

[33] M. Webster‐Clark , R. K. Ross , A. P. Keil , and R. W. Platt , “Variable Selection When Estimating Effects in External Target Populations,” American Journal of Epidemiology 193, no. 8 (2024): 1176–1181. 38629587 10.1093/aje/kwae048 PMC11299018

[34] C. Poole , I. Shrier , and T. J. VanderWeele , “Is the Risk Difference Really a More Heterogeneous Measure?,” Epidemiology 26, no. 5 (2015): 714–718. 26196684 10.1097/EDE.0000000000000354

[35] M. Webster‐Clark and A. P. Keil , “How Choice of Effect Measure Influences Minimally Sufficient Adjustment Sets for External Validity,” American Journal of Epidemiology 192, no. 7 (2023): 1148–1154. 36813295 10.1093/aje/kwad041

[36] G. Sarri , E. Patorno , H. Yuan , et al., “Framework for the Synthesis of Non‐Randomised Studies and Randomised Controlled Trials: A Guidance on Conducting a Systematic Review and Meta‐Analysis for Healthcare Decision Making,” BMJ Evidence Based Medicine 27, no. 2 (2022): 109–119. 10.1136/bmjebm-2020-111493 PMC8961747 33298465

[37] G. Sarri , D. Bennett , T. Debray , et al., “ISPE‐Endorsed Guidance in Using Electronic Health Records for Comparative Effectiveness Research in COVID‐19: Opportunities and Trade‐Offs,” Clinical Pharmacology and Therapeutics 112, no. 5 (2022): 990–999. 35170021 10.1002/cpt.2560 PMC9087010

[38] EMA , “Guideline on Registry‐Based Studies,” European Medicines Agency, 2021.

[39] EMA , “European Medicines Agencies Network Strategy to 2025,” European Medicines Agency, 2025.

[40] NICE , “NICE Real‐World Evidence Framework,” National Institute for Health and Care Excellence, 2022. 10.57264/cer-2023-0135 PMC10690376 37855246

[41] CDA‐AMC , Guidance for Reporting Real‐World Evidence (Canadian Agency for Drugs and Technologies in Health, 2023).

[42] ENCePP , “ENCePP Guide on Methodological Standards in Pharmacoepidemiology,” 2025, https://encepp.europa.eu/encepp‐toolkit/methodological‐guide_en.

[43] S. V. Wang and A. Pottegård , “Advancing Research Transparency and Reproducibility in Pharmacoepidemiology,” Pharmacoepidemiology and Drug Safety 34, no. 2 (2025): e70096. 39948333 10.1002/pds.70096

[44] D. Westreich , J. K. Edwards , C. R. Lesko , E. Stuart , and S. R. Cole , “Transportability of Trial Results Using Inverse Odds of Sampling Weights,” American Journal of Epidemiology 186, no. 8 (2017): 1010–1014. 28535275 10.1093/aje/kwx164 PMC5860052

[45] I. J. Dahabreh , S. J. A. Haneuse , J. M. Robins , et al., “Study Designs for Extending Causal Inferences From a Randomized Trial to a Target Population,” American Journal of Epidemiology 190, no. 8 (2021): 1632–1642. 33324969 10.1093/aje/kwaa270 PMC8536837

[46] S. V. Ramagopalan , S. Popat , A. Gupta , et al., “Transportability of Overall Survival Estimates From US to Canadian Patients With Advanced Non‐Small Cell Lung Cancer With Implications for Regulatory and Health Technology Assessment,” JAMA Network Open 5, no. 11 (2022): e2239874. 36326765 10.1001/jamanetworkopen.2022.39874 PMC9634498

[47] R. J. Cook and J. F. Lawless , “Statistical and Scientific Considerations Concerning the Interpretation, Replicability, and Transportability of Research Findings,” Journal of Rheumatology 51, no. 2 (2024): 117–129. 37967911 10.3899/jrheum.2023-0499

[48] I. J. Dahabreh , S. E. Robertson , L. C. Petito , M. A. Hernán , and J. A. Steingrimsson , “Efficient and Robust Methods for Causally Interpretable Meta‐Analysis: Transporting Inferences From Multiple Randomized Trials to a Target Population,” Biometrics 79, no. 2 (2023): 1057–1072. 35789478 10.1111/biom.13716 PMC10948002

[49] M. R. Elliott , O. Carroll , R. Grieve , and J. Carpenter , “Improving Transportability of Randomized Controlled Trial Inference Using Robust Prediction Methods,” Statistical Methods in Medical Research 32, no. 12 (2023): 2365–2385. 37936293 10.1177/09622802231210944

[50] E. Tipton , “How Generalizable Is Your Experiment? An Index for Comparing Experimental Samples and Populations,” Journal of Educational and Behavioral Statistics 39, no. 6 (2014): 478–501.

[51] R. J. Cook and J. F. Lawless , “Statistical and Scientific Considerations Concerning the Interpretation, Replicability, and Transportability of Research Findings,” J Rheumatol 151, no. 2 (2024): 117–129. 10.3899/jrheum.2023-0499 37967911

[52] E. Tipton , “How Generalizable Is Your Experiment? An Index for Comparing Experimental Samples and Populations,” Journal of Educational and Behavioral Statistics 39, no. 6 (2014): 478–501.

