# Supplementary material for: Transportability Analyses in Comparative Effectiveness Research: A Conceptual Framework and Methodological Principles Endorsed by the International Society for Pharmacoepidemiology
Source: Pharmacoepidemiol Drug Saf. 2026 May 17;35:e70396. doi: 10.1002/pds.70396 (PMC13180502; doi:10.1002/pds.70396)
Supplement: Supplementary file 1 — Table S1: Studies identified in literature review. Figure S2: Potential product life‐cycle applications for transportability exercises in comparative‐effectiveness research. [file PDS-35-e70396-s001.docx]

**SUPPLEMENTARY MATERIALS**

**Transportability Analyses in Comparative Effectiveness Research: A Conceptual Framework and Methodological Principles** **Endorsed by the International Society for Pharmacoepidemiology**

Authors: Blythe Adamson, PhD MPH^1,2^, Wei Liu^3^, Stephen Duffield^4^, Anouk Déruaz- Luyet^5^, Dimitri Bennett^6^, Montse Soriano Gabarró^7^, Mehmet Burcu^8^, Daniela Claudia Moga^9^, Sarah Welby^10^, Tianze Jiao^11^, Xuerong Wen^12^, Grammati Sarri^13^

**Affiliations**

^1^ The Comparative Health Outcomes, Policy and Economics Institute, School of Pharmacy, University of Washington, Seattle, WA, USA

^2^ Infectious Economics, LLC, New York, NY, USA

^3*^ Office of Surveillance and Epidemiology, Center for Drug Evaluation and Research, US Food and Drug Administration, Silver Spring, Maryland, USA

^4^ National Institute for Health and Care Excellence, UK

^5^ Boehringer Ingelheim International GmbH, Germany

^6^ Takeda Development Center Americas, Inc. Cambridge, MA, USA

^7^ Independent researcher, Potsdam, Germany

^8^ Merck & Co., Inc., Rahway, NJ, USA

^9^ University of Kentucky, Lexington, KY, USA

^10^ GSK, Wavre, Belgium

^11^ University of Florida, Gainesville, FL, USA

^12^ University of Rhode Island, Kingston, Rhode Island, USA

^13^ Cytel, Inc, London, UK

*At the time this manuscript was developed.

**SUPPLEMENTARY TABLE S1 Studies identified in literature review**

| **Author, year** | **Title** | **Publication type** |
| --- | --- | --- |
| [Cook RR 2024](https://aithor.com/paper-summary/statistical-and-scientific-considerations-concerning-the-interpretation-replicability-and-transportability-of-research-findings) | Statistical and scientific considerations concerning the interpretation, replicability, and transportability of research findings | Review |
| Cook RR 2024 | Selection processes, transportability, and failure time analysis in life history studies | Methodology |
| Dahabreh IJ 2023C* | Sensitivity analysis using bias functions for studies extending inferences from a randomized trial to a target population | Methodology |
| Dahabreh IJ 2024 | Using trial and observational data to assess effectiveness: trial emulation, transportability, benchmarking, and joint analysis. | Methodology |
| Dahabreh IJ 2024 | Learning about treatment effects in a new target population under transportability assumptions for relative effect measures. | Review |
| Degtiar I 2023 | Conditional cross-design synthesis estimators for generalizability in Medicaid | Methodology |
| Elliott MR 2023* | Improving transportability of randomized controlled trial inference using robust prediction methods | Methodology |
| Fehr 2022 | Assessing the transportability of clinical prediction models for cognitive impairment using causal models. | Real Case Study |
| Gupta 2024 | Transportability of patient outcomes from a US clinical trial to real-world populations - a case study using Lung-MAP S1400I (NCT02785952) | Real Case Study |
| Inoue K 2021*^ | Generalizability and transportability of the national lung screening trial data: Extending trial results to different populations | Methodology |
| Josey KP 2021* | Transporting experimental results with entropy balancing | Methodology |
| Josey KP 2022* | A calibration approach to transportability and data-fusion with observational data | Methodology |
| Kent (2024) | Evaluating transportability of overall survival estimates from US to UK populations receiving first-line treatment for advanced non-small cell lung cancer: a retrospective cohort study. | Real Case Study |
| Levy (2024) | Use of transportability methods for real-world evidence generation: a review of current applications | Review |
| Manke-Reimers F 2025 | When, why and how are estimated effects transported between populations? A scoping review of studies applying transportability methods | Review |
| Mollan KR 2021*^ | Transportability from randomized trials to clinical care: On initial HIV treatment with efavirenz and suicidal thoughts or behaviors | Case Study |
| Ramagopalan SV 2022*^ | Transportability of overall survival estimates from us to canadian patients with advanced non-small cell lung cancer with implications for regulatory and health technology assessment | Case Study |
| Robertson SE 2024A* | Comparing lung cancer screening strategies in a nationally representative us population using transportability methods for the national lung cancer screening trial | Case Study |
| Robertson SE 2024C* | Estimating subgroup effects in generalizability and transportability analyses | Methodology |
| Rudolph KE 2020* | Using transportability to understand differences in mediation mechanisms across trial sites of a housing voucher experiment | Case Study |
| Turner 2024* | Transporting Comparative Effectiveness Evidence Between Countries: Considerations for Health Technology Assessments. | Methodology |
| Vuong 2025 | Systematic review of applied transportability and generalizability analyses: A landscape analysis. | Review |
| Webster-Clark 2025 | Transporting: What Is It and How Do You Do It? | Review |
| Wei L 2023 | Transportability of two heart failure trials to a disease registry using individual patient data | Case study |
| Zivich PN 2024* | Transportability without positivity: A synthesis of statistical and simulation modelling | Methodology |
| Zuo S 2022* | Transportability methods for time-to-event outcomes: Application in adjuvant colon cancer trials | Case study |

*Studies identified by the review of Vuong et al, 2025 ^Studies identified by the review of Levy et al, 2024


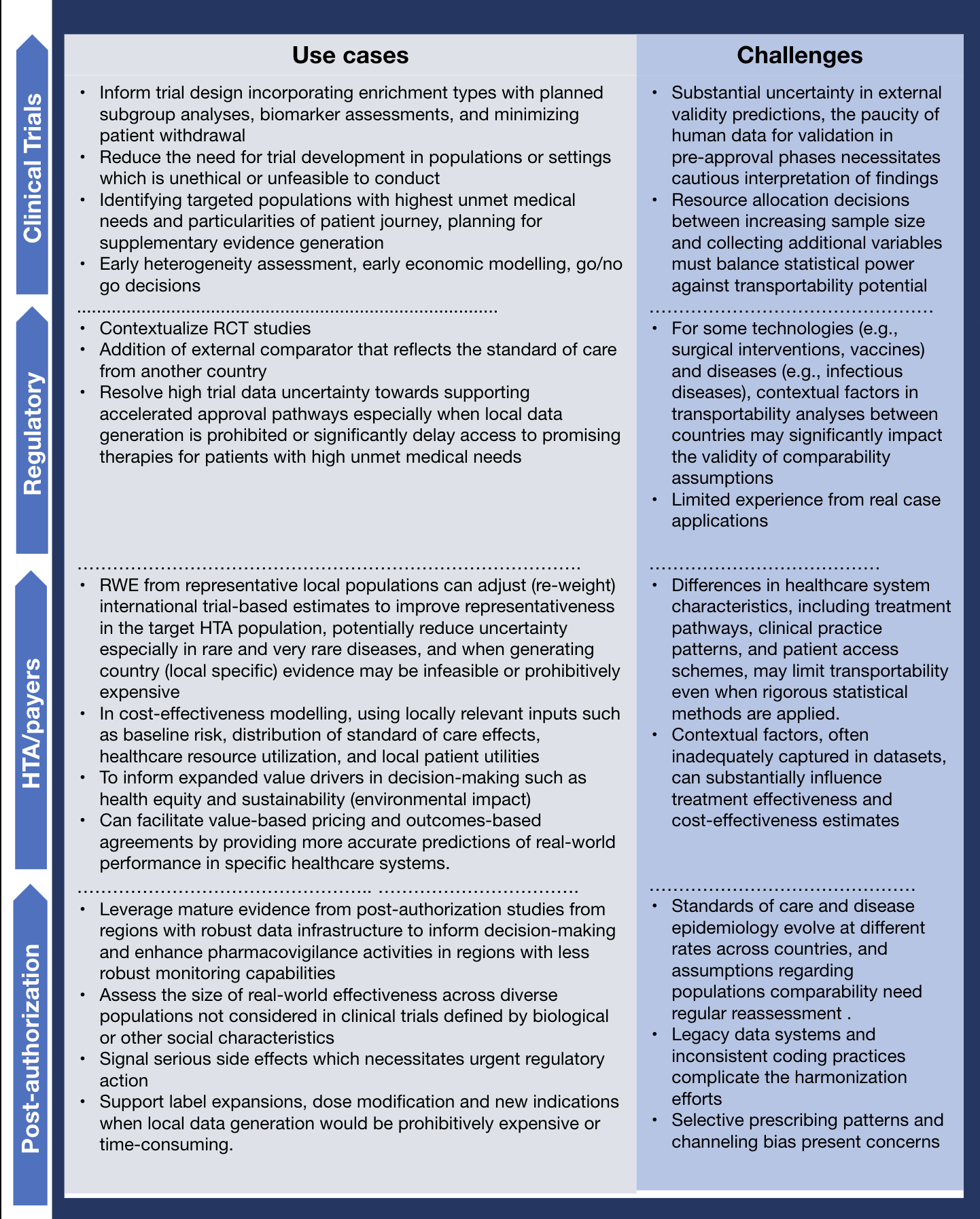


**FIGURE S2.** Potential product life-cycle applications for transportability exercises in comparative-effectiveness research

1. Berger ML, Sox H, Willke RJ, et al. Good practices for real-world data studies of treatment and/or comparative effectiveness: Recommendations from the joint ISPOR-ISPE Special Task Force on real-world evidence in health care decision making. *Pharmacoepidemiol Drug Saf*. Sep 2017;26(9):1033-1039. doi:10.1002/pds.4297

2. Franklin JM, Liaw KL, Iyasu S, Critchlow CW, Dreyer NA. Real-world evidence to support regulatory decision making: New or expanded medical product indications. *Pharmacoepidemiol Drug Saf*. Jun 2021;30(6):685-693. doi:10.1002/pds.5222

3. Yuan H, Ali MS, Brouwer ES, et al. Real-World Evidence: What It Is and What It Can Tell Us According to the International Society for Pharmacoepidemiology (ISPE) Comparative Effectiveness Research (CER) Special Interest Group (SIG). *Clin Pharmacol Ther*. Aug 2018;104(2):239-241. doi:10.1002/cpt.1086

4. FDA. *Considerations for generating clinical evidence from oncology multiregional clinical development programs; draft guidance for industry*. September 17, 2025.

5. FDA. *Guidance document. Use of Real-World Evidence to Support Regulatory Decision-Making for Medical Devices. Guidance for Industry and Food and Drug Administration Staff*. 2017. <https://www.fda.gov/regulatory-information/search-fda-guidance-documents/use-real-world-evidence-support-regulatory-decision-making-medical-devices>

6. EMA. *Guideline on registry-based studies*. 2021. <https://www.ema.europa.eu/en/documents/report/european-union-medicines-agencies-network-strategy-2025-protecting-public-health-time-rapid-change_en.pdf>

7. EMA. *European medicines agencies network strategy to 2025*. 2025. <https://www.ema.europa.eu/en/documents/report/european-union-medicines-agencies-network-strategy-2025-protecting-public-health-time-rapid-change_en.pdf>

8. NICE. *NICE real-world evidence framework*. 2022. <https://www.nice.org.uk/corporate/ecd9/resources/nice-realworld-evidence-framework-pdf-1124020816837>

9. CDA-AMC. *Guidance for Reporting Real-World Evidence*. 2023. <https://www.cadth.ca/sites/default/files/RWE/MG0020/MG0020-RWE-Guidance-Report-Secured.pdf>

10. ENCePP. ENCePP Guide on Methodological Standards in Pharmacoepidemiology. European Network of Centres for Pharmacoepidemiology and Pharmacovigilance. <https://encepp.europa.eu/encepp-toolkit/methodological-guide_en>

11. Wang SV, Pinheiro S, Hua W, et al. STaRT-RWE: structured template for planning and reporting on the implementation of real world evidence studies. *Bmj*. Jan 12 2021;372:m4856. doi:10.1136/bmj.m4856

12. Wang SV, Pottegård A, Crown W, et al. HARmonized Protocol Template to Enhance Reproducibility of hypothesis evaluating real-world evidence studies on treatment effects: A good practices report of a joint ISPE/ISPOR task force. *Pharmacoepidemiol Drug Saf*. Jan 2023;32(1):44-55. doi:10.1002/pds.5507

13. Hernán MA, VanderWeele TJ. Compound treatments and transportability of causal inference. *Epidemiology*. May 2011;22(3):368-77. doi:10.1097/EDE.0b013e3182109296

14. Dahabreh IJ, Robertson SE, Steingrimsson JA, Stuart EA, Hernán MA. Extending inferences from a randomized trial to a new target population. *Stat Med*. Jun 30 2020;39(14):1999-2014. doi:10.1002/sim.8426

15. Pearl J, Bareinboim E. External validity: from do-calculus to transportability across populations. *Stat Sci*. 2014;29(4):579-595.

16. Steckler A, McLeroy KR. The importance of external validity. *Am J Public Health*. Jan 2008;98(1):9-10. doi:10.2105/ajph.2007.126847

17. Degtiar I, Rose S. A review of generalizability and transportability. *Annu Rev Stat Appl*. 2022;10(1):501-524. doi:10.1146/annurev-statistics-042522-103837

18. Gupta A, Chan K, Gomes M, et al. Transportability of patient outcomes from a US clinical trial to real-world populations-a case study using Lung-MAP S1400I (NCT02785952). *medRxiv*. 2024;27:2024-2025.

19. Policy DMIfH. International Harmonization of Real World Evidence Standards Dashboard. Accessed April 16, 2025. <https://healthpolicy.duke.edu/projects/international-harmonization-real-world-evidence-standards-dashboard>

20. EFPIA. *60,000 fewer clinical trial places for Europeans, despite global surge in research projects*. January 1, 2025.

21. Webster-Clark M, Breskin A, Duchesneau ED, Rudolph KE. Transporting: What Is It and How Do You Do It? *Curr Epidemiol Rep*. 2025;12doi:10.1007/s40471-025-00374-6

22. Jaksa A, Arena PJ, Chan KK, Ben-Joseph RH, Jónsson P, Campbell UB. Transferability of real-world data across borders for regulatory and health technology assessment decision-making. *Front Medicine*. 2022;16(9):1073678. doi:10.3389/fmed.2022.1073678

23. Levy NS, Arena PJ, Jemielita T, et al. Use of transportability methods for real-world evidence generation: a review of current applications. *J Comp Eff Res*. Nov 2024;13(11):e240064. doi:10.57264/cer-2024-0064

24. Vuong Q, Metcalfe RK, Ling A, Ackerman B, Inoue K, Park JJ. Systematic review of applied transportability and generalizability analyses: A landscape analysis. *Ann Epidemiol*. Apr 2025;104:61-70. doi:10.1016/j.annepidem.2025.03.001

25. Turner AJ, Sammon C, Latimer N, et al. Transporting Comparative Effectiveness Evidence Between Countries: Considerations for Health Technology Assessments. *Pharmacoeconomics*. Feb 2024;42(2):165-176. doi:10.1007/s40273-023-01323-1

26. Scelo G, Zugna D, Popovic M, Strandberg-Larsen K, Richiardi L. Transporting results in an observational epidemiology setting: purposes, methods, and applied example. *Front Epidemiol*. 2024;4:1335241. doi:10.3389/fepid.2024.1335241

27. Kent S, Mpofu P, Duffield S, et al. Evaluating transportability of overall survival estimates from US to UK populations receiving first-line treatment for advanced non-small cell lung cancer: a retrospective cohort study. *BMJ Open*. Dec 7 2024;14(12):e085722. doi:10.1136/bmjopen-2024-085722

28. Manke-Reimers F, Brugger V, Bärnighausen T, Kohler S. When, why and how are estimated effects transported between populations? A scoping review of studies applying transportability methods. *Eur J Epidemiol*. Mar 2025;40(3):255-273. doi:10.1007/s10654-025-01217-w

29. ICH. *ICH Harmonised Guideline: Addendum on Estimands and Sensitivity Analysis in Clinical Trials to the Guideline on Statistical Principles for Clinical Trials, ICH E9(R1)* 2019. <https://database.ich.org/sites/default/files/E9-R1_Step4_Guideline_2019_1203.pdf>

30. Remiro-Azócar A. Transportability of model-based estimands in evidence synthesis. *Stat Med*. Sep 30 2024;43(22):4217-4249. doi:10.1002/sim.10111

31. Chen J, Scharfstein D, Wang H, et al. Estimands in real-world evidence studies. *Stat Biopharma Res*. 2023;16:1-25. doi:10.1080/19466315.2023.2259829

32. Hernán MA, Robins JM. *Causal inference: what if*. Chapman & Hall/CRC; 2020.

33. VanderWeele TJ, Hernán MA. Causal Inference Under Multiple Versions of Treatment. *J Causal Inference*. May 1 2013;1(1):1-20. doi:10.1515/jci-2012-0002

34. Rudolph KE, Williams NT, Stuart EA, Díaz I. Improving efficiency in transporting average treatment effects. *Biometrika*. 2025;112(3)doi:10.1093/biomet/asaf027

35. Webster-Clark M, Ross RK, Keil AP, Platt RW. Variable selection when estimating effects in external target populations. *Am J Epidemiol*. Aug 5 2024;193(8):1176-1181. doi:10.1093/aje/kwae048

36. Poole C, Shrier I, VanderWeele TJ. Is the Risk Difference Really a More Heterogeneous Measure? *Epidemiology*. Sep 2015;26(5):714-8. doi:10.1097/ede.0000000000000354

37. Webster-Clark M, Keil AP. How Choice of Effect Measure Influences Minimally Sufficient Adjustment Sets for External Validity. *Am J Epidemiol*. Jul 7 2023;192(7):1148-1154. doi:10.1093/aje/kwad041

38. Dahabreh IJ, Haneuse SJA, Robins JM, et al. Study Designs for Extending Causal Inferences From a Randomized Trial to a Target Population. *Am J Epidemiol*. Aug 1 2021;190(8):1632-1642. doi:10.1093/aje/kwaa270

39. Dahabreh IJ, Robertson SE, Petito LC, Hernán MA, Steingrimsson JA. Efficient and robust methods for causally interpretable meta-analysis: Transporting inferences from multiple randomized trials to a target population. *Biometrics*. Jun 2023;79(2):1057-1072. doi:10.1111/biom.13716

40. Elliott MR, Carroll O, Grieve R, Carpenter J. Improving transportability of randomized controlled trial inference using robust prediction methods. *Stat Methods Med Res*. Dec 2023;32(12):2365-2385. doi:10.1177/09622802231210944

41. Sarri G, Patorno E, Yuan H, et al. Framework for the synthesis of non-randomised studies and randomised controlled trials: a guidance on conducting a systematic review and meta-analysis for healthcare decision making. *BMJ Evid Based Med*. Apr 2022;27(2):109-119. doi:10.1136/bmjebm-2020-111493

42. Sarri G, Bennett D, Debray T, et al. ISPE-Endorsed Guidance in Using Electronic Health Records for Comparative Effectiveness Research in COVID-19: Opportunities and Trade-Offs. *Clin Pharmacol Ther*. Nov 2022;112(5):990-999. doi:10.1002/cpt.2560

43. RECORD. What is RECORD? Reporting of studies Conducted using Observational Routinely-collected Data. <https://www.record-statement.org/>

44. Wang SV, Pottegård A. Advancing Research Transparency and Reproducibility in Pharmacoepidemiology. *Pharmacoepidemiol Drug Saf*. Feb 2025;34(2):e70096. doi:10.1002/pds.70096

45. Westreich D, Edwards JK, Lesko CR, Stuart E, Cole SR. Transportability of Trial Results Using Inverse Odds of Sampling Weights. *Am J Epidemiol*. Oct 15 2017;186(8):1010-1014. doi:10.1093/aje/kwx164

46. Lund JL, Webster-Clark MA, Hinton SP, Shmuel S, Stürmer T, Sanoff HK. Effectiveness of adjuvant FOLFOX vs 5FU/LV in adults over age 65 with stage II and III colon cancer using a novel hybrid approach. *Pharmacoepidemiol Drug Saf*. Dec 2020;29(12):1579-1587. doi:10.1002/pds.5148

47. Ramagopalan SV, Popat S, Gupta A, et al. Transportability of Overall Survival Estimates From US to Canadian Patients With Advanced Non-Small Cell Lung Cancer With Implications for Regulatory and Health Technology Assessment. *JAMA Netw Open*. Nov 1 2022;5(11):e2239874. doi:10.1001/jamanetworkopen.2022.39874

48. Cook RJ, Lawless JF. Statistical and Scientific Considerations Concerning the Interpretation, Replicability, and Transportability of Research Findings. *J Rheumatol*. Feb 1 2024;51(2):117-129. doi:10.3899/jrheum.2023-0499

49. Tipton E. How generalizable is your experiment? An index for comparing experimental samples and populations. *J Educ Behav Stat*. 2014;39(6):478-501. doi:10.3102/1076998614558486

50. Signorovitch JE, Wu EQ, Yu AP, et al. Comparative effectiveness without head-to-head trials: a method for matching-adjusted indirect comparisons applied to psoriasis treatment with adalimumab or etanercept. *Pharmacoeconomics*. 2010;28(10):935-45. doi:10.2165/11538370-000000000-00000

51. Josey KP, Berkowitz SA, Ghosh D, Raghavan S. Transporting experimental results with entropy balancing. *Stat Med*. Aug 30 2021;40(19):4310-4326. doi:10.1002/sim.9031

52. Bareinboim E, Pearl J. A general algorithm for deciding transportability of experimental results. *J Causal Inference*. 2013;1(1):107-134. doi:10.1515/jci-2012-0004
